# Diffraction evidence for the structure of cellulose microfibrils in bamboo, a model for grass and cereal celluloses

**DOI:** 10.1186/s12870-015-0538-x

**Published:** 2015-06-23

**Authors:** Lynne H. Thomas, V. Trevor Forsyth, Anne Martel, Isabelle Grillo, Clemens M. Altaner, Michael C. Jarvis

**Affiliations:** Department of Chemistry, University of Bath, Claverton Down, Bath, BA2 7AY UK; Institut Laue-Langevin, Grenoble, Cedex 9 38042 France; EPSAM/ISTM, Keele University, Staffordshire, ST5 5BG UK; New Zealand School of Forestry, University of Canterbury, Christchurch, 4180 New Zealand; School of Chemistry, Glasgow University, Glasgow, G12 8QQ UK

**Keywords:** WAXS, WANS, SANS, Crystallinity, Aggregation, Cellulase

## Abstract

**Background:**

Cellulose from grasses and cereals makes up much of the potential raw material for biofuel production. It is not clear if cellulose microfibrils from grasses and cereals differ in structure from those of other plants. The structures of the highly oriented cellulose microfibrils in the cell walls of the internodes of the bamboo *Pseudosasa amabilis* are reported. Strong orientation facilitated the use of a range of scattering techniques.

**Results:**

Small-angle neutron scattering provided evidence of extensive aggregation by hydrogen bonding through the hydrophilic edges of the sheets of chains. The microfibrils had a mean centre-to-centre distance of 3.0 nm in the dry state, expanding on hydration. The expansion on hydration suggests that this distance between centres was through the hydrophilic faces of adjacent microfibrils. However in the other direction, perpendicular to the sheets of chains, the mean, disorder-corrected Scherrer dimension from wide-angle X-ray scattering was 3.8 nm. It is possible that this dimension is increased by twinning (crystallographic coalescence) of thinner microfibrils over part of their length, through the hydrophobic faces. The wide-angle scattering data also showed that the microfibrils had a relatively large intersheet *d*-spacing and small monoclinic angle, features normally considered characteristic of primary-wall cellulose.

**Conclusions:**

Bamboo microfibrils have features found in both primary-wall and secondary-wall cellulose, but are crystallographically coalescent to a greater extent than is common in celluloses from other plants. The extensive aggregation and local coalescence of the microfibrils are likely to have parallels in other grass and cereal species and to influence the accessibility of cellulose to degradative enzymes during conversion to liquid biofuels

## Background

Cellulose comprises long microfibrils, each a few nm in diameter and containing some tens of glucan chains. The structure of cellulose microfibrils, partially crystalline and partially disordered, is not fully known [1]. Cellulose from cereal crop residues and from grasses like *Miscanthus* is a sustainable starting point for biofuels [2] and, increasingly, for bio-based chemical manufacturing [3]. The conversion of cellulose to useful products can be achieved by enzymatic depolymerisation [4] and is inhibited by lignification, by incompletely understood features of microfibril structure and by aggregation of the microfibrils [5,6].

Evidence has emerged, first from ^13^C NMR spectroscopy [7-9] and more recently from other spectroscopic and scattering technologies [10-15], for partially ordered cellulose microfibrils no more than about 3 nm in diameter. Cellulose microfibrils of that size have been reported from unlignified primary cell walls [13,15] and from gymnosperm xylem, which is dominated by lignified secondary cell walls [7,10,16], although cotton, flax and certain other materials composed of relatively pure cellulose contain thicker microfibrils [14,17,18]. A 3 nm microfibril is too thin to accommodate the 36 chains formerly assumed to be present in microfibrils emerging from the 6-membered ‘rosette’ responsible for cellulose biosynthesis [19]. Recently, based on spectroscopic and scattering evidence, partially ordered 18- and 24-chain models have been suggested for mung bean, celery and spruce wood cellulose [10,13,15]. In primary cell walls, microfibrils of approximately this size may be stacked or ‘twinned’ along part of their length, cohering through the hydrophobic [200] crystal face so that the mean lateral dimension is slightly increased in that direction [15,20]. An 18-chain microfibril model with some ‘twinning’ of this nature appeared to fit the X-ray and NMR data for mung bean primary-wall cellulose [13]. It is not clear whether similar microfibril structures are present in grass and cereal celluloses dominated by lignified secondary walls, for which the most detailed recent model is the flattened-hexagonal, 36-chain structure proposed on AFM evidence for the cellulose of corn stover [19].

It would therefore be of interest to examine the structure of cellulose microfibrils in a grass or cereal species, using the scattering methods that have led to models with less than 36 chains for the microfibrils of non-graminaceous plants. A technical problem is that some of these methods require very well-oriented microfibrils [15]. Highly uniform cellulose orientation is not a well-established feature of most grass and cereal tissues. Bamboo cellulose, however, is particularly well-oriented [21,22]. This feature is responsible for the high stiffness of some bamboo species [22], and its adoption as an engineering material both as intact canes and as the fibre component in biocomposites [23]. In other respects bamboos are typical, if overgrown, grasses [24,25]. Here we report evidence for cellulose microfibril structure in the commercially important bamboo species *Pseudosasa amabilis* (Tonkin cane).

## Results

### Wide-angle X-ray scattering (WAXS)

**Fig. 1:**
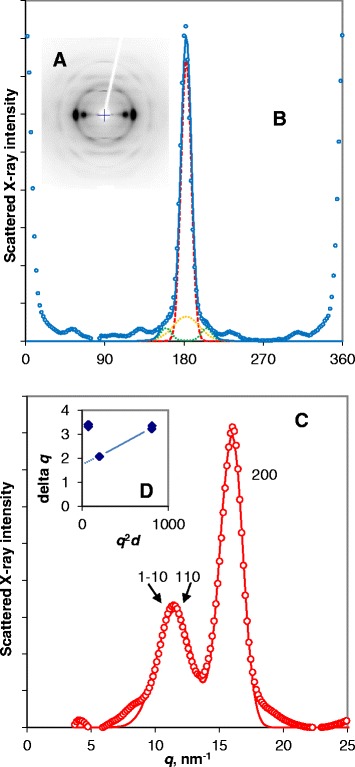
**a** WAXS pattern from bamboo cellulose using Cu K*α* radiation. The fibre axis is vertical. **b** Microfibril orientation from the azimuthal distribution of the 200 reflection. Dotted lines show fitted wide and narrow components. **c** Background-corrected equatorial reflections. **d** Plot of integral width *δq* against *q* ^2^ *d* for the principal equatorial reflections. The integral widths of the 1–10 and 110 reflections lie well above the line projected through the integral widths of the 200 and 400 reflections

Intact internode tissue from mature bamboo stems gave a well-oriented fibre diffraction pattern (Figs. 1a and a). In the azimuthal direction it was possible to dissect the orientation distribution into a wide and a narrow component (Fig. 1a), corresponding perhaps to different cell-wall layers [21] or to different cell types within the vascular bundles. In the radial direction, the background-corrected equatorial profile obtained with Cu Kα radiation is shown in Fig. 1c. It resembled that observed [22] for bamboo cellulose and had some similarities to the corresponding profile for spruce wood [10]. However the 200 reflection was narrower and at slightly lower *q* than for spruce wood implying a mean intersheet spacing (0.403 nm +/− 0.001 nm from three diffraction patterns using both Cu and Mo radiation) about 3 % wider than in spruce cellulose. The 1–10 and 110 reflections were strongly overlapped, implying a smaller monoclinic angle than in wood or in the published cellulose Iβ structure [26]. The mean best-fit monoclinic angle was 92°, although this parameter was difficult to estimate because broadening and overlap of the 1–10 and 110 reflections made them hard to distinguish from one another. The wide intersheet spacing and small monoclinic angle match the observations of Driemeier et al. [27] on sugar cane cellulose.

Wide intersheet spacing and a small monoclinic angle are features normally associated with primary-wall celluloses [13,15,28], but the radial width of the equatorial reflections from bamboo cellulose was considerably less than has been observed from primary-wall celluloses, indicating either greater crystallite dimensions or less disorder. Separating the disorder-related and size-related components of broadening as described by [10,14] gave a Scherrer dimension (mean column length) of 3.84 nm ± 0.13 nm (*n* = 3) perpendicular to the [200] lattice plane and a value of 0.036 ± 0.001 for the disorder parameter *g*. This value of *g* is in agreement with other cellulosic materials but the Scherrer dimension is greater than was found for spruce wood or primary-wall cellulose [10,14]. The [200] Scherrer dimension calculated here was also greater than was estimated previously for bamboo cellulose [22], as expected because of the allowance made here for disorder-related broadening. Broadening of the 1–10 and 110 reflections was difficult to quantify because of the strong overlap between them and because their broadening appeared to be less asymmetric than that of the 200 reflection. With the best-fit value of the monoclinic angle they were clearly substantially wider at half height than the 200 reflection, implying shorter dimensions and/or higher disorder in these crystallographic directions.

An unusual feature of the equatorial scattering profile from this well-oriented bamboo cellulose was the presence of a weak 100 shoulder close to *q* = 8 nm^−1^, which might indicate an anomaly in intersheet stagger, or the spacing between alternate sheets of chains exposed at a [010] face of the microfibril.

### Wide-angle neutron scattering (WANS)

**Fig. 2:**
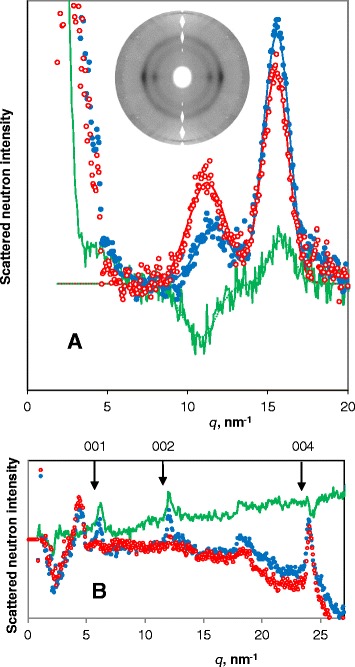
WANS pattern from bamboo cellulose, with and without deuteration. **a** Background-corrected equatorial reflections. Inset: the two-dimensional WANS pattern from bamboo in the H form. The fibre axis is vertical. **b** Reflections on the fibre axis. Closed circles: D form. Open circles: H form. Thin line: difference D-H. Dotted line: fitted equatorial profile

Wide-angle neutron scattering patterns were recorded from bamboo with and without prior equilibration with D_2_O to exchange surface hydroxyl groups. In cellulose Iβ, complete deuteration (which requires much more extreme conditions) slightly increases the relative intensity of the 200 reflection and greatly decreases the relative intensity of the 1–10 reflection [26]. Since the cellulose Iβ lattice is too close-packed to be permeable to H_2_O or D_2_O, any difference between the H and D diffraction patterns (Fig. 2) may be concluded to be derived from hydroxyl groups that were accessible to D_2_O and located either at the surface of the microfibrils, or in disordered internal regions, or in any hemicellulose segments that might be ordered enough to adopt the same chain conformation as cellulose.

The 200 reflection was at essentially the same position before and after deuteration, so that the difference diffraction pattern (Fig. 2) showed only the increase in intensity. The width of the 200 reflection was slightly less than was observed by WAXS implying, if anything, a slightly greater Scherrer dimension perpendicular to the sheets of chains. However the absence of a 400 reflection with measureable intensity in WANS prevented the calculation of a disorder correction.

The negative value of the 1–10 reflection (*q* = 11 nm^−1^) in the D-H difference diffraction pattern allowed its position to be established and differentiated from the overlapping 110 reflection. Fitting the H and D diffraction patterns on the hypothesis that the 1–10 and 110 reflections were unaltered in *q* by deuteration, the best-fit spacing implied a monoclinic angle of 94°, in reasonable agreement with the best-fit value of the monoclinic angle from WAXS. The equatorial part of the WANS pattern was thus consistent with the same lateral *d*-spacings for the domains accessible to deuteration as for the inaccessible domains, implying a surface location for the majority of the deuteration. D_2_O-accessible regions within the microfibrils, if abundant, would require looser chain packing which was not observed.

The signal:noise ratio in WANS was insufficient for the 100 reflection to be distinguished. On the fibre axis, the 001 and 002 reflections were observed only after deuteration (Fig. 2b), implying that there was some irregularity in the longitudinal stagger of the accessible chains exposed at the surfaces of the microfibrils.

### Small-angle neutron scattering (SANS)

**Fig. 3:**
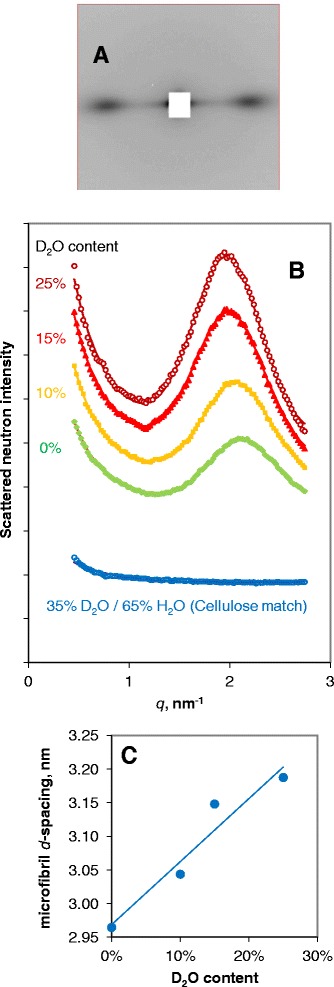
SANS of bamboo cellulose, hydrated to varying extents with D_2_O. **a** Two-dimensional scattering pattern at 25 % D_2_O. The fibre axis is vertical. **b** Radial distribution of equatorial SANS intensity as a function of D_2_O content, with small-angle Bragg peak in the region of *q* = 2 nm^−1^. **c** Effect of hydration with D_2_O on the *d*-spacing between microfibrils, calculated from the *q* value of the Bragg peak

When cellulose microfibrils aggregate together with any regularity, Bragg scattering (diffraction) at small angles can be observed from the arrayed microfibrils themselves, in addition to the wide-angle scattering from the crystal planes within the microfibrils [12]. In woody materials if the microfibrils are in close contact, there will be insufficient matrix material between them to provide the contrast for small-angle Bragg scattering of X-rays. However if the microfibrils can be forced apart by D_2_O there is intense neutron scattering contrast between the D_2_O and the cellulose, as can be seen at low *q* in Fig. 2a. Starting from bamboo saturated with D_2_O, the D_2_O content was progressively reduced to zero in the absence of H_2_O. Considerable SANS contrast remained at zero D_2_O content (Fig. 3a) due to exchange of hydroxyl groups on cellulose surfaces [15] or hemicelluloses. As the D_2_O content was reduced the small-angle Bragg peak moved to higher *q*, implying that on drying the nominal centre-to-centre spacing of the microfibrils narrowed from 3.19 nm at 25 % D_2_O to 2.96 nm at 0 % D_2_O (Fig. 3c). It may be assumed that the centre-to-centre spacing at 0 % D_2_O corresponds to microfibrils touching one another and is therefore equal to the microfibril diameter. After drying the remaining deuterium atoms were on hydroxyl groups, not water molecules. It is therefore likely that it was contact through the hydrophilic faces of the microfibrils that gave rise to the small-angle Bragg scattering, not through the 200 faces suggested as the sites of microfibril coalescence (twinning).

No small-angle Bragg peak was observed from bamboo equilibrated with 35 % D_2_O: 65 % H_2_O. A mixture of D_2_O and H_2_O in these proportions matches the cellulose scattering length density and thus gives zero contrast between the liquid phase and cellulose [12]. This observation showed that the spacing observed was indeed between cellulose microfibrils, not lignin or some other feature of the cell-wall structure of bamboo, such as arabinoxylans. The *d*-spacings shown in Fig. 3b do not necessarily correspond to any form of global mean, because the scattering contrast is likely to be greatest when the microfibrils are just far enough apart to permit D_2_O to enter between them: wider spacings are probably too irregular for strong Bragg scattering. The Bragg peaks observed in D_2_O were broad, indicating that only a few microfibrils were packed laterally together, or that the packing was disordered, or most probably both.

## Discussion

The wide-angle and small-angle scattering patterns and NMR spectra for bamboo cellulose resembled those from wood and dicot primary cell walls in many respects, but there were interesting differences. Although bamboo internodes can certainly be called woody, with secondary wall layers and strong lignification [21] the unit cell parameters of the crystalline cellulose fraction resembled those of primary cell walls, with a small monoclinic angle and relatively large intersheet [200] *d*-spacing. Essentially the same intersheet *d*-spacing was measured by neutron scattering when the accessible cellulose chains were deuterated. This observation strongly suggests that most of the D_2_O-accessible cellulose chains were at the microfibril surface rather than buried in the interior, since the chain packing appeared to be as tight as in other crystalline celluloses into which water cannot penetrate.

**Fig. 4:**
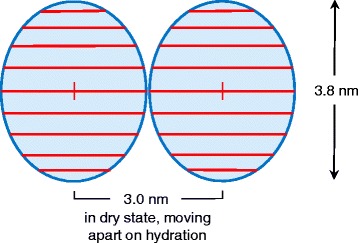
Proposed average dimensions for microfibrils of bamboo cellulose, from WAXS (vertical dimension) and SANS (horizontal spacing). Each of the microfibrils is shown with the (200) lattice plane, corresponding to the orientation of the sheets of hydrogen-bonded chains, horizontal. The elliptical shape of the microfibrils as shown is merely diagrammatic, avoiding assumptions about which lattice planes are exposed at the surface

The diameters of cellulose microfibrils have often been estimated on the assumption that they are approximately as wide as they are high [10], although the AFM study of Ding and Himmel [19] suggested that maize primary-wall microfibrils were about 3 nm high perpendicular to the [200] plane and 3.6 nm wide parallel to the [200] plane. The different techniques used here provide information on microfibril dimensions in each lateral direction. The Scherrer dimension obtained by WAXS after disorder correction was 3.8 nm perpendicular to the [200] plane, and the WANS data implied that 3.8 nm was not an overestimate in this direction. Bamboo microfibrils, therefore, are substantially larger in this dimension, on average, than microfibrils of softwood [10] or dicot primary-wall cellulose [13,15]. The WAXS data suggested smaller lateral dimensions in other directions, but this inference was not quantitative because the disorder correction was difficult to apply to broadening of the 1–10 and 110 reflections. The mean centre-to centre distance of 3.0 nm, estimated from the position of the SANS coherent scattering peak, must include hydrogen-bonding cellulose surfaces that deuterate to provide the SANS contrast. This distance cannot therefore be perpendicular to the [200] plane; it could be parallel or diagonal to that plane depending on which crystal faces form the boundaries of the microfibril. A mean sheet width of three chains, giving a mean dimension of about 3 nm in that direction, would be consistent with the WAXS data on the assumption that there was substantial disorder at the hydrophilic faces of the microfibril Fig. 4.

A cellulose chain within the Iβ crystal structure occupies 0.32 nm^2^ in cross-section [26] or 0.33 nm^2^ with the slightly larger *d*-spacings found for bamboo. This cross-sectional area would suggest that the observed microfibril dimensions, 3.8 nm perpendicular to the sheets of chains and 3.0 nm across the sheets, would allow space for about 34 chains. However the irregular hydrophilic surfaces of the microfibrils mean that fewer chains can be fitted within these overall dimensions. Based on microfibril models similar to those suggested for spruce cellulose [10] the number of chains would be about 26–30 depending on the detailed shape of the microfibrils. That would be consistent with the 18-chain model proposed for mung bean primary-wall cellulose [13] only if there were a much greater extent of ‘stacking’ or ‘twinning’ in which two 18-chain microfibrils coalesce through the [200] faces for part of their length. The suggested dimensions and this pattern of coalescence and divergence recall the AFM observations by Ding and Himmel [19] on the microfibrils of maize primary cell walls, but with the crystal lattice turned through 90°. AFM methods give no indication of the orientation of the lattice planes. It should be stressed that only averaged dimensions can be derived from our data, and the dimension obtained by SANS is not a true average. The data of Wang et al. [22] on local crystallographic variability between bamboo cell walls, the developmental variation in maize recorded by Zhang et al. [29] and the intricate aggregation of maize microfibrils imaged by Ding and Himmel [19], show that averaged dimensions may conceal complex local patterns of variation. The ‘twinning’ or ‘stacking’ (crystallographic coalescence) phenomenon proposed by Newman et al. [13] and Thomas et al. [15] may be sufficient to provide a large part of this variation without assuming heterogeneity in the structures of microfibrils extruded by the terminal complexes that carry out their biosynthesis [30].

Aggregation of cellulose microfibrils in bamboo and in other monocotyledonous species [19,22] appears to involve contact with and without crystalline coalescence. How such aggregation interferes with the access of cellulases to the cellulose surfaces that they attack, and how chemical pretreatments impact on the extent of microfibril aggregation [6], are questions that deserve closer attention during the development of enzymatic processes for manufacturing biofuels and bio-based materials from grass and cereal biomass.

## Conclusions

The microfibrils of bamboo cellulose, although derived mainly from secondary cell walls, resembled the primary-wall celluloses of other plants in having relatively wide inter-sheet spacing and small monoclinic angle. The mean microfibril diameter was 3.8 nm perpendicular to the sheets of chains, unusually large for a woody material but consistent with fusion of pairs of smaller microfibrils over part of their length. The bamboo microfibrils were also loosely aggregated into bundles with a limited degree of regularity in spacing. D_2_O was able to penetrate into the microfibril bundles, increasing the microfibril spacing as hydration progressed.

## Methods

### Material

Tonkin cane (*Pseudosasa amabilis*) internodes were split and the interior removed to leave strips of the outer tissue approximately 2 mm wide × 1 mm deep.

### Small-angle neutron scattering (SANS)

SANS analysis was conducted on the high-flux beamline D33 at the Institut Laue-Langevin (ILL), Grenoble. The neutron beam had a wavelength λ = 3.5 Å with spread Δλ/λ = 10 %, and was passed through a 2.8 m long collimator tube. Sample-to-detector distance was 2 m. The *q* range covered in this experiment extended from 0.4 nm^−1^ to 2.8 nm^−1^. A number of bamboo segments about 1 mm thick were placed side by side to give a sheet wider than the beam diameter. The bamboo segments were saturated with H_2_O, D_2_O or 35:65 D_2_O:H_2_O, the contrast match composition for cellulose, and then equilibrated with phosphorus pentoxide to dry to a predetermined weight. The samples were immediately sealed in an aluminium foil package 15 mm square. At least 1 h was then allowed for internal equilibration of moisture [10]. An empty foil container was used as background.

### Wide-angle X-ray scattering (WAXS)

X-ray diffraction patterns were obtained at ambient temperature using a Rigaku R-axis/RAPID image plate diffractometer. Both Cu Kα (λ = 0.15406 nm, one sample) and Mo (λ = 0.7071 nm, two samples) sources were used, with the beam collimated to a diameter of 0.5 mm. Scattering angles were expressed as *q* = 4πsinθ/λ. Samples were 1 mm thick in the direction parallel to the beam and their other dimensions exceeded the beam diameter. The diffraction patterns were collected in perpendicular transmission mode. Radial profiles of scattered intensity I as a function of *q* were integrated over azimuthal angles of 2° using the AreaMax software package (Rigaku/MSC, Tokyo). Background correction was carried out as described [10]. Each tangential profile was fitted by a dual Gaussian function and the narrower of the two Gaussians was used to reconstruct the equatorial radial profile [14]. In the radial direction, the overlapping 1–10 and 110 reflections were fitted by two Gaussian functions and the 200 reflection was fitted by an asymmetric function *F*(*q*) constructed as follows: when *q* > the point of maximum intensity *q*_0_, *F*(*q*) = *F*_0_(*q*), a simple Gaussian function. When *q* < *q*_0_, *F*(*q*) = *F*_0_(*q*)(1 + 0.1(*q* - *q*_0_)^2^). It was assumed that the integral width *δq* of *F*_0_(*q*) was controlled by both disorder and the column length of the crystallite, so that *δq* = *δq*_0_ *+ π/*2 *g*^2^*q*^2^*d,* where *g* is the non-asymmetric disorder parameter and *d* is the lattice spacing. Then a plot of integral width *δq* against *q*^2^*d* is linear with, at the intercept, the Scherrer dimension (mean column length) L = 2*π*/*δq*_0_ [10].

### Wide-angle neutron scattering (WANS)

Bamboo samples were prepared as for SANS at 25 % H_2_O or D_2_O content, sufficient to saturate the cell walls without filling the cell lumina. WANS analysis was conducted on beamline D19 at the ILL. Beamline D19 has a four-circle diffractometer with a cylindrical detector consisting of a 256 × 640 array of gas-filled cells giving an aperture 30° vertically × 120° horizontally. The neutron beam was monochromated to a wavelength of 2.42 Å and the sample-to-detector distance, taken to the electrode plane in each cell at the equator, was 756 mm. The response for each cell of the detector was calibrated using the isotropic incoherent neutron scattering from a vanadium rod, and blank-corrected using an empty aluminium foil container.

The absorption coefficient of the sample along the beam axis was calculated from absorption coefficients based on the elemental composition. Absorption factors at all angles within the aperture of the detector were then calculated using in-house software based on the integrated path length through the sample, which was assumed to have cuboidal geometry and was wider than the neutron beam. The fibre axis was tilted such that the full widths of the 001, 002, 003 and 004 reflections were collected. In-house software was then used to reconstruct the data into reciprocal space and to join together the component images of the diffraction pattern. The combined images were exported into Fit2D, where radial intensity profiles integrated over 10° in azimuth were calculated in the equatorial and meridional directions.

## Abbreviations

NMR: Nuclear Magnetic Resonance
WAXS: Wide-angle X-ray Scattering
WANS: Wide-angle Neutron Scattering
SANS: Small-angle Neutron Scattering
AFM: Atomic Force Microscopy
*gg*: gauche-gauche
*gt*: gauche-trans
*tg*: trans-gauche
